# AI-assisted hemorrhage detection following endovascular stroke treatment: a retrospective diagnostic accuracy study

**DOI:** 10.1186/s42466-026-00457-9

**Published:** 2026-02-03

**Authors:** Luise Endler, Miar Ouaret, Janos Sebestyen Gellén, Johannes A. R. Pfaff

**Affiliations:** 1 Department of Neuroradiology, University Hospital Salzburg Paracelsus Medical University, Ignaz-Harrer-Straße 79, Salzburg, A-5020 Austria; 2 Paracelsus Medical University, Strubergasse 21, 5020 Salzburg, Austria

## Abstract

**Background:**

Antithrombotic therapy is essential for preventing strokes, but its use after reperfusion therapy requires careful monitoring due to the risk of hemorrhagic transformation. Non-contrast-enhanced computed tomography (NCCT) is the standard for detecting intracranial hemorrhages post-stroke. Artificial intelligence may enhance hemorrhage detection and improve patient safety. This study evaluates AI’s sensitivity and specificity in detecting hemorrhagic events in NCCT scans within 48 h after endovascular stroke treatment, compared to standard radiological assessment.

**Methods:**

A retrospective, single-center study was conducted at a European stroke center, including 495 NCCT scans from 425 patients who underwent endovascular stroke treatment between 08/2021 and 06/2024. A CE-marked AI software based on convolutional neural networks (CNN) analyzed the scans independently. The reference standard was assessments of two board-certified neuroradiologists, and AI results were compared with routine radiological reports. Sensitivity, specificity, positive predictive value (PPV), and negative predictive value (NPV) were calculated, and inter-rater reliability was assessed using Cohen’s kappa.

**Results:**

The reference standard identified hemorrhages in 197 NCCT scans. The AI system showed sensitivity of 95.9%, specificity of 84.6%, PPV of 80.4%, and NPV of 96.9%. Radiological reports had sensitivity of 91.9%, specificity of 96.3%, PPV of 94.3%, and NPV of 94.7%. Cohen’s kappa was higher for radiological reports (0.886) than AI (0.780), indicating stronger agreement with the reference standard. AI had a higher false-positive rate (15.4%) than radiological reports (3.7%).

**Conclusions:**

AI demonstrated high sensitivity for detecting intracranial hemorrhages but had a higher false-positive rate compared to routine radiological assessment. While AI can aid clinical decision-making, radiologists show superior overall diagnostic accuracy. Further research is needed to explore the impact of AI-assisted decision-making on stroke management and secondary prevention.

## Introduction

Antithrombotic therapy, including antiplatelet or anticoagulant agents, is recommended for nearly all patients without contraindications to prevent recurrent stroke [1]. However, reperfusion therapies, such as intravenous thrombolysis (IVT) and endovascular thrombectomy (EVT), carry the risk of complications, the most severe being hemorrhagic transformation (HT) [2–4]. 

International guidelines recommend follow-up imaging post-stroke to exclude HT before starting antithrombotic therapy [1]. Non contrast-enhanced computed tomography (NCCT) is the most widely used imaging modality for this purpose. Reliable detection of even small hemorrhages is crucial to prevent potentially life-threatening complications of antithrombotic therapy.

AI-based algorithms demonstrate reliable performance in detecting and segmenting intracranial hemorrhage in non-contrast-enhanced CT scans of acute stroke patients, showing strong agreement with human readers and precise quantification of intraparenchymal hemorrhage volumes [5]. Artificial intelligence (AI) has the potential to assist clinicians in image analysis, enhancing hemorrhage detection and improving patient safety.

Our research aims to evaluate the sensitivity and specificity of AI as a stand-alone tool for detecting hemorrhagic events in NCCT scans performed within 48 h following endovascular stroke treatment, compared to standard radiological assessment in routine clinical practice.

## Methods

We conducted a retrospective, single-center study at a European comprehensive stroke center. This diagnostic accuracy study was designed and reported in accordance with the STARD guidelines [6]. Our study will provide evidence for a critical evaluation of Artificial Intelligence-enabled imaging tools [7]. The study was approved by the local ethics committee and informed consent was waived. All methods were conducted in adherence to ICH/GCP requirements. This study received no external financial support from industry partners.

### Participants

All patients undergoing endovascular stroke treatment for acute ischemic stroke between 08/2021 and 06/2024 were reviewed. The indication and execution of endovascular stroke therapy followed national and international guidelines and recommendations. Inclusion criteria for this study were: (i) endovascular stroke treatment for large or medium vessel occlusion of the anterior or posterior circulation up to the proximal M2- or P2-level, (ii) NCCT images of sufficient quality, i.e., CT scans without severe motion artifacts within 48 h after endovascular stroke treatment. If a patient underwent more than one NCCT scans within 48 h after EVT, each scan was included as a separate case in the analysis. Clinical details of these patients were documented in accordance with ethical approval and are limited to the necessary data for addressing the research question.

### Image acquisition and reconstruction

NCCT imaging was performed using a 64 slice Somatom Definition AS + CT scanner (Siemens Healthineers, Forchheim, Germany) at our comprehensive stroke center. All NCCT scans were performed in an axial plane with 0.6 mm slice thickness. For this clinical routine diagnostics and this study, only axial reformations (soft tissue window) with slice thicknesses of 1 mm and 4 mm were used. These images were archived in the institutional Picture Archiving and Communication System (PACS).

### Test

In accordance with the clinical question and indication for imaging, the primary outcome of the test was to determine whether intracranial hemorrhage was present in the analyzed imaging studies or not. Detected intracranial hemorrhages were classified according to their anatomic description as per Heidelberg Bleeding Classification [8].

All NCCT scans were analyzed using an FDA approved and CE-marked cloud-based AI software, based on a convolutional neural network (CNN) architecture (ICH triage, Aidoc Medical Ltd., Tel Aviv, Israel). The license for the commercial AI system used in this study was purchased from the AI system’s vendor. The AI-generated imaging-evaluations were performed automatically immediately after image acquisition and were recorded separately for this study (AI stand-alone).

Additionally, we collected the radiological reports generated in routine clinical practice by board-certified neuroradiologists (radiological reports). In clinical routine, neuroradiologists had full access to the patient’s medical history, including details of reperfusion therapy, prior imaging, and the AI-generated imaging-evaluations. However, the neuroradiologists were not obligated to use the AI-generated assessments, and it was not documented whether a neuroradiologist considered the AI evaluation when generating their report. Neuroradiologists had the option to override AI findings if they suspected incorrect results. The study design was structured to reflect standard clinical workflows and practices.

The reference standard was set by two board-certified radiologist with 5 and more than 15 years of experience in neuroradiology and unrestricted access to all clinical and imaging data, including information on AI findings, radiological reports, interventional therapy and follow-up. These two radiologists were responsible for establishing ground truth and did not contribute to the imaging assessment and reporting in clinical routine. In case of a discrepancy between the AI evaluation and the radiological reports or one of the reference standard reviewers, NCCTs were reviewed in a joint session until agreement was reached between the reference standard reviewers.

### Statistical analysis

Statistical analyses were performed using IBM SPSS statistics (version 29.0.2.0. (20)). Continuous variables are presented as medians and interquartile intervals, and categorical variables as absolute values and percentages.

Level of agreement of each reader (AI as stand-alone and radiological reports) with the reference standard was evaluated for detection of any intracranial hemorrhage. Inter-rater reliability was analyzed using Cohen’s kappa. A two-sided p-value of 0.05 was considered statistically significant. Sensitivity, specificity, positive predictive value (PPV), and negative predictive value (NPV) were calculated.

## Results

### Study population and imaging data

During the observation period, a total of *n* = 485 patients underwent endovascular stroke treatment at our center. After excluding *n* = 33 patients (*n* = 30 underwent MR imaging only, and *n* = 3 did not receive any brain imaging within 48 h), *n* = 452 patients remained. Each of these patients underwent at least one non-contrast computed tomography (NCCT) within 48 h, and *n* = 78 patients received a second NCCT within the same period, resulting in a total of *n* = 530 NCCT scans available for analysis.

**Fig. 1:**
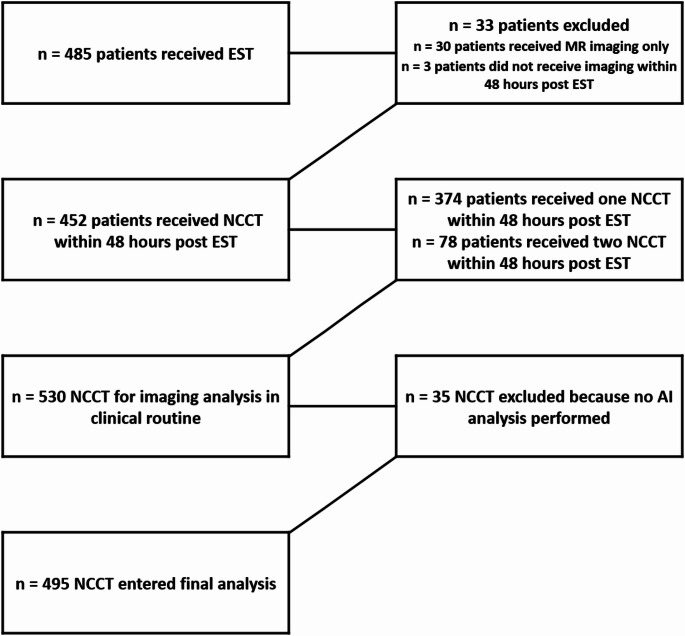
Flowchart of included and excluded patients and studies. AI = artificial intelligence, EST = endovascular stroke therapy, NCCT = Non contrast-enhanced computed tomography, MR = Magnetic resonance imaging

Due to temporary unavailability of the AI system because of maintenance and suspension, *n* = 35 studies from *n* = 27 patients were excluded. No NCCT scans were excluded due to motion artifacts. Ultimately, *n* = 495 NCCT scans from *n* = 425 patients (median age: 77 years [interquartile range: 66–84], female: *n* = 215 [50.6%]) were included in the final analysis (Fig. 1).

### Detection of bleeding events by AI and routine radiological reporting

Among the *n* = 495 analyzed NCCT scans, the reference standard identified *n* = 197 scans as positive for at least one bleeding event. The AI system correctly identified *n* = 189 of these cases, resulting in a false-negative rate of 4.1% and a sensitivity of 95.9%. Additionally, the AI system classified *n* = 46 of the *n* = 298 scans without hemorrhage (according to the reference standard) as positive, leading to a false-positive rate of 15.4% and a specificity of 84.6%. The AI system demonstrated a positive predictive value (PPV) of 80.4% and a negative predictive value (NPV) of 96.9% for the detection of hemorrhagic events.

In routine radiological reporting, *n* = 192 reports explicitly mentioned the presence of hemorrhage. Of these, *n* = 181 were concordant with the reference standard, corresponding to a false-negative rate of 8.1% and a sensitivity of 91.9%. Furthermore, *n* = 11 of the *n* = 298 scans without hemorrhage according to the reference standard were classified as positive in routine radiology reports, yielding a false-positive rate of 3.7% and a specificity of 96.3%. The routine radiological reporting exhibited a PPV of 94.3% and an NPV of 94.7% for hemorrhage detection.

**Fig. 2:**
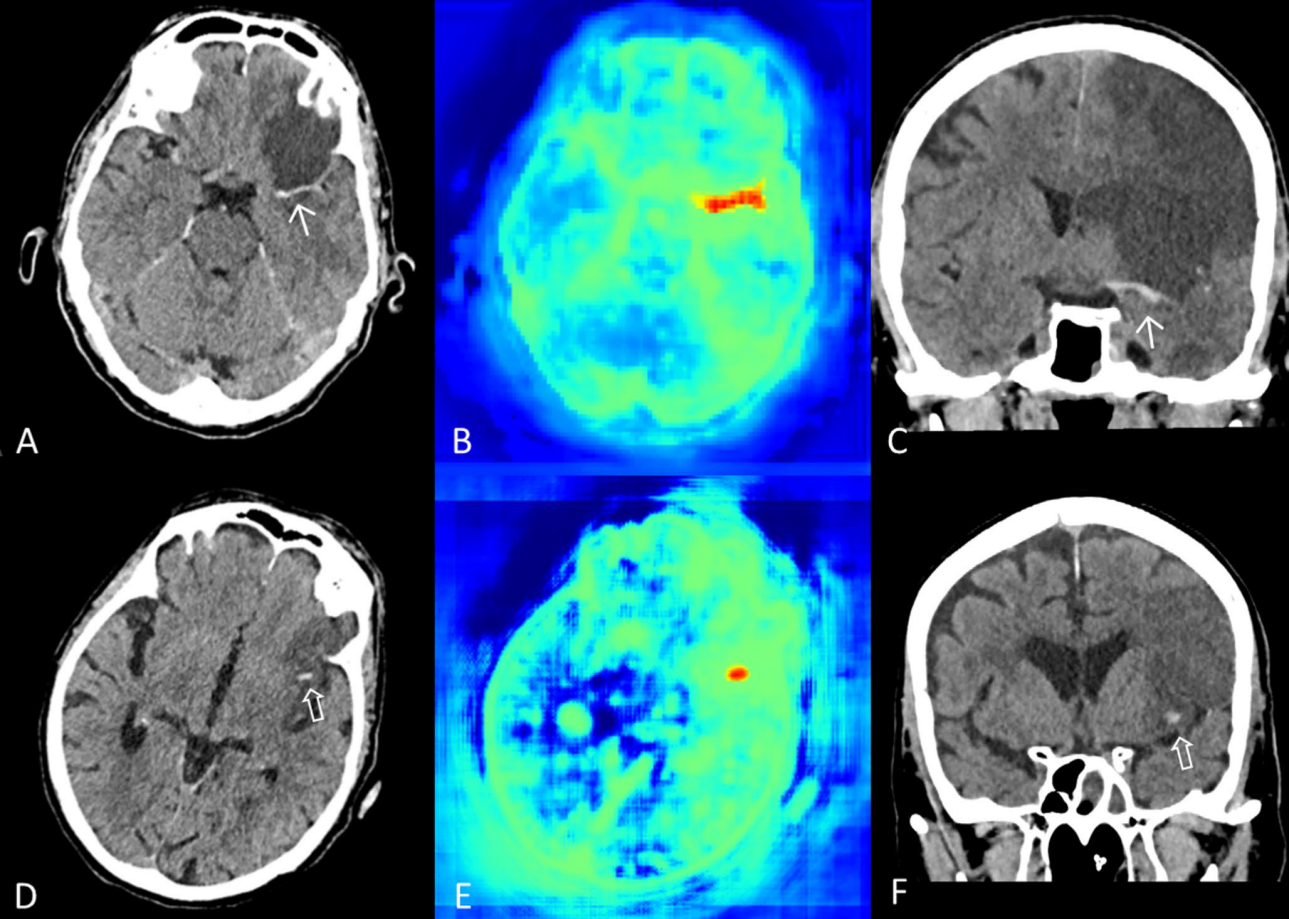
The top row shows a patient after a futile mechanical thrombectomy, with a hyperdense vessel due to a remaining long-segment occlusion of the left carotid-T and middle cerebral artery (see thin white arrows in axial [**A**] and coronal [**C**] non-contrast enhanced CT images), which was falsely identified as hemorrhage by the AI algorithm (**B**), but correctly acknowledged in the radiological report. The bottom row presents a patient after mechanical thrombectomy with a small basal intracerebral hemorrhage (see hollow white arrow in axial [**D**] and coronal [**F**] non-contrast enhanced CT images), which was correctly marked by the AI algorithm (**E**). The radiological report for the patient in the bottom row did not acknowledge the presence of this hemorrhage. Note the similarity in location and configuration of these hyperdense structures in both cases

AI false positive findings were mostly observed when clearly hypodense infarct tissue was located next to tissue or vascular structures with “normal” or slightly increased density. Of the 46 cases that the AI incorrectly classified as hemorrhage (AI false positives), these were identified as vessels (*n* = 19, 41.3%), non-infarcted portions of the brain parenchyma within or adjacent to the infarcted area (*n* = 12, 26.1%), dural sinuses (*n* = 5, 10.9%), cavernomas (*n* = 3, 6.5%), or other typically hyperdense structures in the non-contrast CT, such as the falx, or the tentorium, meningiomas, or large developmental venous anomalies. Among the 11 cases that were incorrectly classified as hemorrhage in the radiological reports, 7 (63.6%) corresponded to non-infarcted brain parenchyma situated within or near the infarcted region. An example illustrating the difficulty in differentiating between cerebral vessels and parenchymal hemorrhages is shown in Fig. 2.

Both the AI system and routine radiological reporting showed a significant correlation with the reference standard (both *p* < 0.001). However, agreement between the AI system and the reference standard, as measured by Cohen’s Kappa (0.780), was lower than that observed for routine radiology reports (0.886).

### Anatomic distribution of bleeding events according to the Heidelberg Bleeding Classification

The anatomical distribution of bleeding events according to the Heidelberg Bleeding Classification is presented in Table 1. The most frequently observed hemorrhages were subarachnoid hemorrhages (HBC class 3c, *n* = 73) and scattered small petechiae without mass effect (HBC class 1a, *n* = 31). These categories also represented the most common types of hemorrhages that were either not detected by the AI system or not described in routine radiological reports (Table 1). Upon review of these cases, it was noted that the missed hemorrhagic events often involved subtle changes that were confirmed, in some cases through hematoma expansion or only through subsequent imaging.

**Table 1:** Distribution of intracranial bleeding events according to the Heidelberg bleeding classification

	Reference Standard		AI		Radiological Reports	
	Number of detected hemorrhages	Percentage of hemorrhages	Number of detected hemorrhages	Percentage of detected hemorrhages	Number of reported hemorrhages	Percentage of reported hemorrhages
Heidelberg Bleeding Classification Class	n	%	n	%	n	%
Total	197	100	189	95.9	181	91.9
1a	31	15.7	26	83.9	26	83.9
1a + 3b + 3c	2	1.0	2	100	2	100
1a + 3c	15	7.7	15	100	14	93.3
1b	19	9.6	19	100	18	94.7
1b + 3c	11	5.6	11	100	10	90.9
1c	9	4.6	9	100	9	100
1c + 3a + 3c	1	0.5	1	100	1	100
1c + 3b + 3c	6	3.0	6	100	6	100
1c + 3c	15	7.6	15	100	15	100
2 + 3b + 3c	5	2.5	5	100	5	100
2 + 3c	4	2.0	4	100	4	100
3a + 3b + 3c	1	0.5	1	100	1	100
3a + 3c	1	0.5	1	100	1	100
3b + 3c	1	0.5	1	100	1	100
3c	73	37.1	70	95.9	67	91.8
3d	3	1.5	3	100	1	33.3

## Discussion

This study aimed to evaluate the performance of a commercially available AI-software as a stand-alone tool for detecting intracranial hemorrhagic events in NCCT scans performed within 48 h following endovascular stroke treatment, compared to standard radiological assessment. Our findings indicate that the AI system demonstrated a high sensitivity (95.9%) but a lower specificity (84.6%) compared to routine radiological assessment (sensitivity: 91.9%, specificity: 96.3%). The AI system had a higher false-positive rate (15.4%) compared to radiological reporting (3.7%), suggesting a tendency toward overcalling hemorrhages. Conversely, the AI system had a lower false-negative rate (4.1%) than routine reporting (8.1%), indicating strong performance in detecting hemorrhages. However, agreement between AI and the reference standard was lower than for routine radiological reports, highlighting that radiologists maintained a higher level of diagnostic accuracy in clinical practice.

Our findings align with existing studies demonstrating the utility of AI in radiological diagnostics, often achieving expert-level performance [5, 9, 10]. Schmitt et al. report, for example, that in a selected patient cohort including patients with stroke symptoms the AI ​​detection of intracranial hemorrhage had a sensitivity and specificity for ICH detection of 0.91 and 0.89 [5]. In contrast to previous studies focusing on hemorrhage detection in emergent care or trauma patients [5, 11–14], our study extends prior work by evaluating AI in a different specialized setting – stroke patients following mechanical thrombectomy. The AI system performed well despite the altered hospital environment and differing patient demographics compared to its original development and validation settings. However, our results also reaffirm the critical role of human radiologists in clinical practice.

The clinical relevance of our findings becomes evident when analyzing the distribution of detected and missed hemorrhages according to the Heidelberg Bleeding Classification. The AI system effectively flagged all NCCT scans with more than one intracranial hemorrhagic event and all patients with hemorrhagic transformation of the infarcted tissue that exceeded small petechiae without mass effect. This is particularly relevant for two reasons: Patients with intracranial hemorrhages after endovascular stroke treatment are prone to worse clinical outcome [15, 16], and all patients required subsequent antithrombotic therapy, including antiplatelet or anticoagulant agents, to prevent recurrent strokes unless contraindicated, as a measure of secondary prevention.

Early (re-)initiation of anticoagulation therapy is associated with improved outcomes but is only considered safe if no intracranial hemorrhage is present in the infarcted area following reperfusion therapy [17, 18]. According to inclusion and exclusion criteria from the Early Versus Late Initiation of Direct Oral Anticoagulants in Post-ischaemic Stroke Patients With Atrial fibrillatioN (ELAN) trial, hemorrhagic infarcts classified as HI1 or HI2 according to the European Cooperative Acute Stroke Study (ECASS) classification (corresponding to classes 1a and 1b of the Heidelberg Bleeding Classification [19]) do not necessarily contraindicate early initiation of direct oral anticoagulants, provided no clinical deterioration occurs [20]. This suggests that even if the AI would miss class 1a hemorrhages, i.e. scattered small petechiae without mass effect, the impact on early anticoagulation initiation might be minimal [21].

For antiplatelet therapy, our results are also meaningful. Current guidelines recommend initiating antiplatelet therapy as soon as possible after imaging excludes hemorrhage, ideally within 12 to 24 h of symptom onset [1, 22]. Recent studies further support early antiplatelet therapy initiation within 24 h following endovascular stroke treatment [23].

However, for subarachnoid hemorrhages (SAH), data on early treatment initiation of antithrombotic therapy is limited. SAH is a common and potentially serious complication following EVT, and management strategies remain debated [24]. The clinical approach may depend on hemorrhage extent and etiology, such as vessel perforation during the procedure or avulsion of small perforators due to arterial straightening during retrieval of a thrombectomy device.

Ultimately, the decision to and the timing of (re-)initiation of antithrombotic therapy after an ischemic stroke inherently carries risks that must be carefully weighed up and it is up to each treatment team how proactively they implement this therapy. An example for the application of AI as a stand-alone diagnostic tool before starting or restarting antithrombotic therapy after an ischemic stroke would be in situations where immediate image review by a radiologist is unavailable or impractical—such as healthcare settings without on-site radiologists (teleradiology) or during temporary staff shortages—while still aiming to begin antithrombotic treatment as promptly as possible. Although our data point favorably in this direction, the data are not yet sufficient to establish this in clinical routine.

### Limitations

While our study provides valuable insights, it has several limitations. First, it was conducted at a single comprehensive stroke center, which may limit the generalizability of our findings to other institutions with different imaging protocols and AI implementation strategies. Second, our study was retrospective, and prospective validation is necessary to further assess the clinical impact of AI in real-world settings, especially with regard to secondary prevention in stroke patients.

Finally, while we compared AI to radiologists in routine clinical practice, we did not evaluate its performance against blinded radiologists, which might have provided additional insights. In the case of blinded radiologists, our data would probably only change on the radiological reporting side, since the AI evaluated the image data as a stand-alone. The study design deliberately compared AI against routine clinical radiological assessments, where multiple radiologists with access to patient history and treatment data interpreted scans. Despite this inherent imbalance, the AI system demonstrated a strong stand-alone performance. A direct comparison with radiologists blinded to clinical data would not reflect real-world application, as diagnostic decisions in clinical practice rely on integrated patient information. However, we acknowledge that the fact that radiologists had the opportunity to consult the AI’s evaluation during their reporting also represents a possible source of bias. Additionally, unlike AI, which evaluates each image on its own without knowledge of previous examinations, radiologists are subject to a certain degree of bias when making their reports due to comparison with previous examinations and findings.

## Conclusion

This study demonstrates that AI can achieve high sensitivity in detecting intracranial hemorrhages in non contrast-enhanced computed tomography scans following endovascular stroke treatment. However, its higher false-positive rate indicates a tendency for overcalling hemorrhages. Radiologists maintain superior overall diagnostic accuracy over AI as stand-alone imaging evaluation. The clinical impact of AI-assisted decision-making on stroke management and secondary prevention strategies is promising, but warrants further investigation.

## Data Availability

All data generated or analysed during this study are included in this published article.

## Abbreviations

AI: Artificial intelligence
ECASS: European Cooperative Acute Stroke Study
EVT: endovascular thrombectomy
HBC: Heidelberg Bleeding Classification
HT: hemorrhagic transformation
IVT: intravenous thrombolysis
NCCT: Non-contrast-enhanced computed tomography
SAH: subarachnoid hemorrhage
